# Genetic variants and haplotypes in fibulin-5 (*FBLN5*) are associated with pseudoexfoliation glaucoma but not with pseudoexfoliation syndrome

**DOI:** 10.1042/BSR20221622

**Published:** 2023-03-02

**Authors:** Ramani Shyam Kapuganti, Barsha Bharati, Pranjya Paramita Mohanty, Debasmita Pankaj Alone

**Affiliations:** 1 School of Biological Sciences, National Institute of Science Education and Research (NISER) Bhubaneswar, P.O. Bhimpur-Padanpur, Jatni, Khurda, Odisha 752050, India; 2 Homi Bhabha National Institute (HBNI), Training School Complex, Anushaktinagar, Mumbai, 400094, India; 3 Sri Sri Borda Hospital, Dhauli, Bhubaneswar, Odisha 751002, India

**Keywords:** fibulin-5, functional variant, pseudoexfoliation glaucoma, tag genotyping

## Abstract

Pseudoexfoliation (PEX) is a multifactorial age-related disease involving deposition of extracellular proteinaceous aggregates on anterior ocular tissues. The present study aims to identify functional variants in fibulin-5 (*FBLN5*) as risk factors for the development of PEX. Thirteen tag single-nucleotide polymorphisms (SNPs) in *FBLN5* were genotyped using TaqMan SNP genotyping technology to identify association between SNPs of *FBLN5* and PEX in an Indian cohort comprising 200 control and 273 PEX patients (169 PEXS and 104 PEXG). Functional analysis of risk variants was done through luciferase reporter assays and electrophoretic mobility shift assay (EMSA) using human lens epithelial cells. Genetic association and risk haplotype analysis showed a significant association of rs17732466:G>A (NC_000014.9:g.91913280G>A) and rs72705342:C>T (NC_000014.9:g.91890855C>T) within *FBLN5* as risk factors with the advanced severe stage of the disease, pseudoexfoliation glaucoma (PEXG). Reporter assays showed allele-specific regulatory effect of rs72705342:C>T on gene expression, wherein, construct containing the risk allele showed a significant decrease in the reporter activity compared with the one with protective allele. EMSA further validated higher binding affinity of the risk variant to nuclear protein. *In silico* analysis predicted binding sites for two transcription factors, GR-α and TFII-I with risk allele at rs72705342:C>T, which were lost in the presence of protective allele. The EMSA showed probable binding of both these proteins to rs72705342. In conclusion, the present study identified the novel association of two genetic variants in *FBLN5* with PEXG but not with PEXS, distinguishing between the early and the later forms of PEX. Further, rs72705342:C>T was found to be a functional variant.

## Introduction

Pseudoexfoliation (PEX) is a progressive systemic disease of protein aggregation that involves the deposition of extracellular fibrillar material called PEX fibrils in the tissues of various organs, which manifests prominently in the eye. The initial stage of deposition termed as pseudoexfoliation syndrome (PEXS) progresses to the severe form, pseudoexfoliation glaucoma (PEXG) in almost half of PEXS-affected individuals [1]. The deposits impede the aqueous humour outflow pathways building up the intraocular pressure (IOP), which damages the retinal ganglion cell axons in the optic nerve leading to gradual blindness that are the hallmark features of PEXG [2,3]. PEXS accounts for 25–70% of all open-angle glaucoma cases depending on the country [4]. The prevalence of PEX is highly variable across the globe with 0.0% cases in Eskimos, 1.8% in the United States, 3.8–6.0% in India, 11.0–35.0% in East Africa, 5.0–25.0% in the Scandinavians and 38.0% in Navajo Indians [5–8].

The PEX fibrils or deposits comprise various extracellular proteins, membrane proteins and molecular chaperones, such as elastin, tropoelastin, fibrillin-1, amyloid P, lysyl oxidase like-1, vitronectin, fibulins, clusterin, latent transforming growth factor binding proteins and other components of elastic fibre system, and deregulation of these proteins has been observed at molecular levels in PEX individuals [9–11]. Previously, we reported the down-regulation of the extracellular matrix (ECM) protein, fibulin-5 (FBLN5) in the lens capsule of PEXS individuals [12]. *FBLN5* encodes a secreted extracellular calcium-binding protein highly crucial for assembly of elastic fibres. FBLN5 deposits the cross-linking enzyme, lysyl oxidase-like 1 (LOXL1) in the ECM where it cross-links tropoelastin monomers to elastic fibres and is also involved in other essential cellular functions, such as cell matrix adhesion, integrin-dependent regulation of reactive oxygen species (ROS) in the ECM, regulation of cell receptor signalling, endothelial-to-mesenchymal transition and modulation of matrix proteases [13].

Aberrant expression or deposition of FBLN5 has been observed in age-related macular degeneration (AMD), abdominal aortic aneurysms and pelvic organ prolapse (POP) and individuals with these disorders have shown susceptibility to PEX [14–19]. Further, mutations in *FBLN5* have been related to complications such as AMD, cutis laxa and POP [20–22].

We have previously reported a novel genetic association of two variants, rs7149187 and rs929608 residing in the 5′-UTR and 10th intron, respectively, in *FBLN5* as risk for PEX. However, these variants did not display any functional regulatory role in reporter assays. The main purpose of the present study was to identify putative regulatory variants in *FBLN5* which could be explored further to find out whether they are functional variants playing pivotal role in PEX pathogenesis. In the present study, using a Tag SNP genotyping approach, we identified novel genetic association of two intronic variants in *FBLN5* as risk factors for PEXG, one of which was found to be a functional variant.

## Materials and methods

### Study subjects’ selection and recruitment

The following study was approved by the Institutional Biosafety and Human Ethics committee of National Institute of Science Education and Research and adhered to the tenets of Declaration of Helsinki. Participants were recruited at Sri Sri Borda Hospital, Bhubaneswar, India by our clinical collaborator Dr Pranjya Paramita Mohanty. All the study participants underwent a detailed ocular examination including slit lamp microscopy, ocular biometry, Goldman applanation tonometry, +90D biomicroscopic fundus evaluation and four-mirror gonioscopy. Cataract patients aged above 40 years with clinically evident PEX like material over lens capsule (LC) and pupillary ruff having untreated IOP < 21 mmHg without any visual field defects were included under PEXS group and those with untreated IOP > 21 mmHg, glaucomatous nerve head damage with repeatable field defects corresponding to disc damage were included under PEXG group. Patients with corneal or retinal pathology precluding reliable visual field were excluded from PEXG group. Patients with systemic diseases, such as diabetes, were excluded from the study. Cataract patients aged above 40 years without PEXS or PEXG, with untreated IOP < 21 mmHg having normal discs and visual field were included as controls. Written informed consent was obtained from all participants. Age-sex-matched subjects were chosen for the study.

### SNP selection and TaqMan genotyping

For higher genetic coverage of the *FBLN5* gene, tag SNP genotyping approach was chosen. Thirteen tag SNPs within *FBLN5* were chosen based on 1000 genomes HapMap South Asian GIH dataset with a pair-wise tagging of *r*^2^ > 0.9 and minor allele frequency (MAF) > 0.1. The selected 13 single-nucleotide polymorphisms (SNPs) spanned the *FBLN5* gene and were genotyped in 169 PEXS, 104 PEXG and 200 age-sex matched control subjects (Supplementary Table S1). Peripheral blood was collected from the study subjects and genomic DNA was extracted through phenol-chloroform method. The SNPs were genotyped using TaqMan SNP genotyping assays (Applied Biosystems, Carlsbad, CA, U.S.A.) on Quantstudio 7 (Thermo Fisher Scientific, U.S.A.). Each 5 μl PCR reaction mix consisted of 20 ng DNA sample, 2.5 μl of TaqMan master mix 2X, 1.25 μl of TaqMan SNP assay, and the volume was made up with nuclease-free water. The PCR conditions were initial denaturation at 95°C followed by 40 cycles of denaturation at 95°C for 15 s and annealing and extension at 60°C for 1 min. The data were analysed using the instrument software and TaqMan Genotyper software.

### Cell culture

Human lens epithelial B3 cells (HLE B-3), an immortalized cell line that was derived from the human lens infant tissue and transformed with an adenovirus 12-Simian Virus 40 hybrid (Ad12SV40) [23] were purchased from ATCC (B-3 CRL11421, VA, U.S.A.), grown in DMEM/F12 medium (11330057, Invitrogen GIBCO, U.S.A.) supplemented with 10% inactivated fetal bovine serum (16000044, Invitrogen), 1.0% penicillin (100 U/ml) and streptomycin (0.1 mg/ml) (A001, HiMedia) at 37°C and 5.0% CO_2_.

### Plasmid construction and Luciferase reporter assays

To test the functional effect of the SNPs, two reporter vectors, pGL4.23 with minimal promoter of the reporter and pGL3 containing the FBLN5 core promoter region were used. The region from −675 bp to ATG start codon of FBLN5 gene was amplified using Phusion High Fidelity DNA polymerase (NEB) and cloned into pGL3 basic luciferase reporter vector using BglII and NcoI (NEB). DNA fragments (29 bp long) surrounding the SNPs (Supplementary Table S2) with either of the alleles at the centre were cloned into the reporter vectors using KpnI HF and XhoI (NEB). HLE B-3 cells were seeded in a 24-well plate and at 80% confluency, the cells were transiently transfected with 500 ng of the constructs along with 5 ng of *Renilla* vector (pGL4.74) using lipofectamine (Thermo Fisher Scientific, U.S.A.). After 24-h post transfection, cells were harvested and luciferase activity assayed using Dual-Luciferase Reporter assay system (Promega, U.S.A.). The Firefly luciferase activity from each construct was normalized to Renilla luciferase activity, and the ratio has been plotted as per cent luciferase activity relative to that of empty vector (taken as 100%). Data represent at least three independent experiments.

### Electrophoretic mobility shift assays

A total of 29 base pairs sense (S) and antisense (A) oligonucleotides encompassing the rs72705342:C>T (NC_000014.9:g.91890855C>T) were synthesized for performing the EMSAs (Supplementary Table S2). The oligos were synthesized with their 5′-end labelled with biotin and unlabelled oligos were procured as well. The oligos were annealed by incubating the mix of complementary strands at 95°C for 5 min followed by gradually cooling down the mix to room temperature. Nuclear extract from HLE B-3 cells was prepared using the NE-PER Nuclear and Cytoplasmic Extraction Reagents kit (Thermo Fisher Scientific, U.S.A.). The binding reaction included poly (dI.dC) as non-specific competitor DNA. For competition experiments, a 200-fold excess of unlabelled oligonucleotides was included in the pre-incubation mixture. For supershift assays, EMSA-specific antibodies for TFII I (sc-46670X, Santa Cruz, U.S.A.) and GR-α (PA1516, Invitrogen) were pre-incubated with the nuclear extract for 1 h before adding the final reaction mixture. The complexes after incubation were resolved on 6% native polyacrylamide gels and transferred to nylon membranes and developed. The EMSA was performed with the Lightshift Chemiluminescent EMSA kit (Thermo Fisher Scientific, U.S.A.). Detection was done using Fusion Solo S Chemi-Doc (Vilber Lourmat) and gel shifts were quantified with the Evolution Capt software (Vilber Lourmat Fusion Solo S).

### *In silico* analysis

The PROMO software (http://alggen.lsi.upc.es/cgi-bin/promo_v3/promo/promoinit.cgi?dirDB=TF_8.3 was used to identify transcription factor binding sites to the region surrounding rs72705342C>T. The sequences of genomic region flanking the ‘T’ allele or ‘C’ allele of the SNP rs72705342 (±15 bp) were used as input.

### Genetic and statistical analysis

Age-sex matched samples were taken for the experiments. The matching was done by performing the Student’s *t*-test between the groups. No data were missing for the participants. The allelic association tests, Hardy–Weinberg equilibrium (HWE), and logistic regression analysis for covariates were done using PLINK. Haplotype analysis and linkage disequilibrium (LD) analysis were done using Haploview V4.2. Statistical significance of group-wise results was analysed using Student’s *t*-test and *P*<0.05 was considered as statistically significant. The Bonferroni and Holm correction was applied for multiple pair-wise comparisons. All experiments were done at least three times independently. Data are presented as mean ± SEM.

## Results

### Demographics of the study subjects

A total of 273 PEX (169 PEXS and 104 PEXG) and 200 age-sex matched control subjects participated in the present study. The demographics of the study subjects are shown in Table 1. The mean age in years ± SD of controls, PEXS, and PEXG were 70.17 ± 7.17, 71.09 ± 7.29 and 70.21 ± 7.39, respectively. The age-range of controls, PEXS, and PEXG was 60–90 years, 50–90 years and 60–92 years, respectively. Of the study participants, 36.1% were females. About 171 females (80 control, 64 PEXS and 27 PEXG) and 302 males (120 control, 105 PEXS and 77 PEXG) participated in the study.

**Table 1:** Demographic details of the study subjects

Subjects	Sample size (*N*)	Age (in years)		*P*-values	Sex		*P*-values
		Mean ± SD	Range		Male	Female	
Control	200	70.17 ± 7.17	60–90		120	80	
PEXS	169	71.09 ± 7.29	50–90	0.10	105	64	0.41
PEXG	104	70.21 ± 7.39	60–92	0.50	77	27	0.12

### Intronic variants, rs72705342 and rs17732466, within *FBLN5* are genetically associated with PEXG

**Figure 1:**
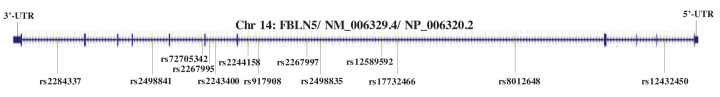
Position of Tag SNPs Gene structure of *FBLN5* showing position of the 13 tag SNPs (obtained from UCSC genome browser June 5, 2022).

Thirteen tag SNPs (Figure 1) within *FBLN5* were genotyped in 169 PEXS, 104 PEXG and 200 age- and sex-matched control subjects. All the studied SNPs passed the HWE test set at a default significance threshold of *P≤0*.001. Allele frequencies, odds ratio (OR) and statistical significance of the genotyped *FBLN5* variants are presented in Table 2. Two variants, NC_000014.9:g.91913280G>A (rs17732466:G>A) and NC_000014.9:g.91890855C>T (rs72705342:C>T) located in the 4th and the 6th introns of *FBLN5*, respectively, were found to be significantly associated with PEXG with the risk alleles being ‘G’ (*P*=0.04) and ‘C’ (*P*=0.02), respectively. Risk analysis showed that the minor alleles ‘A’ at rs17732466 and ‘T’ at rs72705342 confer a protective effect with an *OR* of 0.66 (95% CI: 0.43–0.99) and 0.60 (95% CI: 0.39–0.93), respectively. However, none of the studied variants showed a significant association with PEXS. Genotypic distribution of the variants in controls, PEXS, and PEXG is presented in Table 3. None of the SNPs showed any genotypic association with PEXS. However, individuals with the risk genotype ‘CC’ at rs72705342 showed higher susceptibility to having PEXG compared with individuals carrying the ‘TT’ genotype (*P*=0.04).

**Table 2:** Distribution of *FBLN5* variants in PEXS and PEXG compared with the controls

SNP ID	Major allele	Minor allele	MAF			Control vs. PEXS		Control vs. PEXG	
			Control (*N*=200)	PEXS (*N*=169)	PEXG (*N*=104)	*OR* (95% CI)	*P*-value	*OR* (95% CI)	*P*-value
rs12432450	C	T	0.37	0.39	0.39	1.14 (0.84–1.55)	0.37	1.07 (0.75–1.53)	0.68
rs8012648	C	T	0.35	0.36	0.30	1.04 (0.76–1.41)	0.80	0.77 (0.53–1.12)	0.17
**rs17732466**	G	A	**0.28**	0.26	**0.21**	0.87 (0.62–1.22)	0.44	**0.66 (0.43–0.99)**	**0.04**
rs12589592	G	A	0.25	0.20	0.22	0.78 (0.55–1.12)	0.18	1.03 (0.69–1.53)	0.86
rs2498835	G	T	0.36	0.37	0.30	1.03 (0.75–1.41)	0.74	0.72 (0.50–1.05)	0.08
rs2267997	G	C	0.32	0.32	0.33	1.18 (0.76–1.85)	0.85	1.08 (0.75–1.56)	0.65
rs917908	T	C	0.11	0.13	0.11	0.87 (0.37–2.04)	0.44	0.95 (0.55–1.64)	0.86
rs2244158	C	T	0.31	0.30	0.33	0.94 (0.68–1.30)	0.74	0.98 (0.68–1.43)	0.95
rs2243400	C	T	0.14	0.17	0.15	1.18 (0.78–1.79)	0.40	1.02 (0.62–1.66)	0.93
rs2267995	G	C	0.31	0.31	0.37	1.02 (0.73–1.39)	0.94	1.32 (0.92–1.90)	0.12
**rs72705342**	C	T	**0.25**	0.24	**0.17**	0.96 (0.68–1.35)	0.81	**0.60 (0.39–0.93)**	**0.02**
rs2498841	C	A	0.24	0.26	0.18	1.12 (0.80–1.57)	0.49	0.67 (0.44–1.04)	0.07
rs2284337	G	A	0.29	0.25	0.22	0.84 (0.63–1.17)	0.31	0.71 (0.47–1.07)	0.1

Abbreviations: CI, confidence interval; MAF, minor allele frequency; OR, odds ratio; PEXG, pseudoexfoliation glaucoma; PEXS, pseudoexfoliation syndrome. RefSeq NC_000014.9

**Table 3:** Genotypic distribution of *FBLN5* variants in control, PEXS and PEXG

SNP ID	Genotype	Frequency in control (*n*=200)	Frequency in PEXS (*n*=169)	Frequency in PEXG (*n*=104)	Genetic model	Control vs. PEXS		Control vs. PEXG	
						OR (95% CI)	*P*-value	OR (95% CI)	*P*-value
rs12432450	TT	0.12	0.12	0.05	Additive	1.09 (0.77–1.54)	0.59	1.16 (0.79–1.70)	0.43
	CT	0.51	0.54	0.34	Dominant	1.17 (0.76–1.81)	0.45	1.06 (0.64–1.75)	0.79
	CC	0.37	0.34	0.60	Recessive	1.09 (0.58–2.05)	0.78	1.35 (0.67–2.73)	0.39
rs8012648	TT	0.13	0.15	0.01	Additive	1.05 (0.76–1.44)	0.75	0.81 (0.54–1.21)	0.31
	CT	0.43	0.42	0.34	Dominant	1.02 (0.67–1.55)	0.91	0.79 (0.48–1.29)	0.35
	CC	0.44	0.43	0.65	Recessive	1.11 (0.61–2.02)	0.73	0.71 (0.33–1.55)	0.39
rs17732466	AA	0.10	0.08	0.01	Additive	0.87 (0.59–1.27)	0.47	0.65 (0.39–1.10)	0.11
	AG	0.37	0.35	0.33	Dominant	0.86 (0.56–1.31)	0.49	0.63 (0.38–1.04)	0.07
	GG	0.53	0.56	0.66	Recessive	0.79 (0.37–1.66)	0.53	0.49 (0.18–1.37)	0.17
rs12589592	GG	0.07	0.04	0.13	Additive	0.70 (0.44–1.12)	0.14	0.97 (0.60–1.56)	0.90
	AG	0.35	0.35	0.46	Dominant	0.76 (0.50–1.16)	0.21	1.11 (0.69–1.79)	0.65
	AA	0.58	0.56	0.40	Recessive	0.53 (0.21–1.33)	0.18	0.89 (0.35–2.25)	0.80
rs2498835	TT	0.14	0.15	0.03	Additive	1.05 (0.76–1.44)	0.75	0.657(0.41–1.03)	0.06
	GT	0.43	0.44	0.23	Dominant	1.08 (0.71–1.65)	0.71	0.81 (0.49–1.31)	0.39
	GG	0.43	0.41	0.74	Recessive	1.06 (0.59–1.91)	0.82	0.44 (0.18–1.06)	0.06
rs2267997	CC	0.08	0.13	0.08	Additive	1.22 (0.85–1.76)	0.27	1.10 (0.70–1.73)	0.67
	CG	0.48	0.39	0.45	Dominant	0.86 (0.56–1.31)	0.48	1.10 (0.67–1.80)	0.68
	GG	0.44	0.48	0.46	Recessive	1.72 (0.85–3.46)	0.12	1.16 (0.48–2.75)	0.73
rs917908	CC	0.01	0.04	0.02	Additive	2.66 (0.92–7.74)	0.07	1.94 (0.58–6.52)	0.27
	CT	0.22	0.19	0.18	Dominant	1.04 (0.63–1.72)	0.84	0.88 (0.48–1.60)	0.68
	TT	0.77	0.77	0.80	Recessive	7.27 (0.86–61.0)	0.06	3.95 (0.35–44.20)	0.26
rs2244158	TT	0.07	0.09	0.09	Additive	1.10 (0.74–1.65)	0.61	1.06 (0.66–1.71)	0.79
	CT	0.49	0.41	0.49	Dominant	0.82 (0.54–1.25)	0.36	0.92 (0.56–1.50)	0.75
	CC	0.44	0.49	0.42	Recessive	1.39 (0.64–3.03)	0.39	1.20 (0.48–3.00)	0.69
rs2243400	TT	0.01	0.03	0.07	Additive	1.76 (0.77–4.05)	0.17	1.69 (0.68–4.11)	0.25
	CT	0.27	0.27	0.45	Dominant	1.13 (0.71–1.80)	0.59	0.93 (0.53–1.61)	0.80
	CC	0.72	0.69	0.48	Recessive	3.08 (0.59–16.12)	0.18	2.96 (0.48–18.0)	0.23
rs2267995	CC	0.09	0.09	0.06	Additive	0.98 (0.67–1.43)	0.92	1.29 (0.86–1.93)	0.20
	CG	0.42	0.44	0.38	Dominant	1.05 (0.69–1.60)	0.80	1.37 (0.83–2.24)	0.20
	GG	0.48	0.47	0.55	Recessive	0.93 (0.44–1.93)	0.85	1.47 (0.68–3.13)	0.31
**rs72705342**	TT	0.07	0.06	0.05	**Additive**	0.93 (0.60–1.45)	0.77	**0.35 (0.12–0.98)**	**0.04**
	CT	0.37	0.36	0.31	Dominant	0.93 (0.61–1.41)	0.74	0.64 (0.39–1.06)	0.08
	CC	0.56	0.58	0.64	Recessive	0.90 (0.38–2.11)	0.81	0.14 (0.01–1.08)	0.05
rs2498841	AA	0.04	0.05	0.10	Additive	1.18 (0.69–2.00)	0.53	0.48 (0.16–1.40)	0.18
	AC	0.40	0.42	0.40	Dominant	1.12 (0.74–1.69)	0.58	0.68 (0.41–1.12)	0.13
	CC	0.56	0.53	0.49	Recessive	1.34 (0.47–3.78)	0.57	0.26 (0.03–2.21)	0.22
rs2284337	AA	0.09	0.04	0.15	Additive	0.67 (0.42–1.07)	0.09	0.69 (0.41–1.17)	0.17
	AG	0.39	0.42	0.49	Dominant	0.93 (0.61–1.41)	0.73	0.69 (0.42–1.14)	0.15
	GG	0.52	0.54	0.36	Recessive	0.45 (0.18–1.11)	0.08	0.54 (0.19–1.52)	0.24

Abbreviations: CI, confidence interval; OR, odds ratio; PEXG, pseudoexfoliation glaucoma; PEXS, pseudoexfoliation syndrome. RefSeq NC_000014.9.

### Genetic association of haplotypes was observed with PEXG

**Figure 2:**
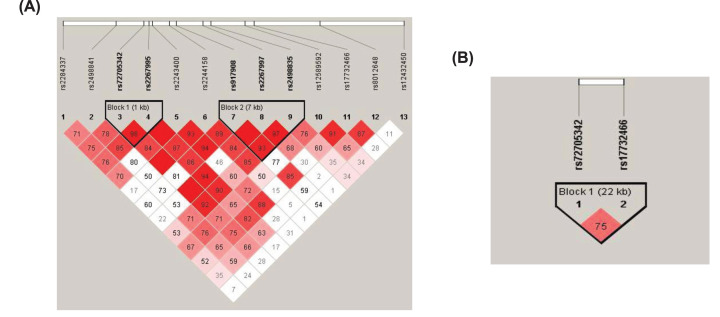
Linkage disequilibrium pattern and haplotype association analysis (**A**) LD pattern across all the SNPs. The LD blocks are defined using the confidence interval algorithm. (**B**) LD block pattern with rs72705342-rs17732466.

The LD pattern across the 13 studied *FBLN5* SNPs is shown in Figure 2A. The confidence interval algorithm (Gabriel et al*.*) defined two LD blocks. The frequency of haplotype ‘T-G’ in Block 1 (rs72705342-rs2267995) was found to be significantly lower in PEXG (0.17, *P*=0.03) but not in PEXS (0.23, *P*=0.70) compared with control (0.24) (Table 4). None of the haplotypes in Block 2 (rs917908-rs2267997-rs2498835) showed association with either PEXS or PEXG (Supplementary Table S3). Also, the haplotype analysis was done for the SNPs that were significantly associated with PEXG (Figure 2B). The frequency of haplotype ‘C-G’ at ‘rs72705342-rs17732466’ was significantly higher in PEXG (0.76, *P*=0.03) compared with controls (0.67) but was not associated with PEXS (0.69, *P*=0.6, Table 4).

**Table 4:** Haplotype association of *FBLN5* variants with PEXS and PEXG

Haplotype ‘T-G’ (rs72705342-rs2267995)	Frequency	OR (95% CI)	*P*-value
Control	0.24	1.06 (0.75–1.50)	0.70
PEXS	0.23		
**PEXG**	**0.17**	**0.63 (0.41–0.98)**	**0.03**

Haplotype ‘G-C’ (rs17732466-rs72705342)	Frequency	OR (95% CI)	*P*-value
Control	0.67	0.93 (0.68–1.28)	0.69
PEXS	0.69		
**PEXG**	**0.76**	**1.51 (1.02–2.22)**	**0.03**

### rs72705342 shows allele-specific regulatory effect

**Figure 3:**
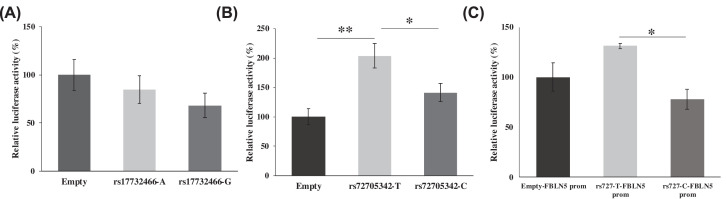
Putative regulatory effect of *FBLN5* variants (**A**) Relative luciferase activity of constructs containing rs17732466 with either ‘A’ or ‘G’ alleles cloned upstream of the minimal promoter. No difference in reporter activities was observed with either ‘A’ (84.8 ± 14.3) or ‘G’ (68.2 ± 12.9) alleles compared with empty vector (100.0 ± 15.8) with *P*-values 0.50 and 0.17, respectively. Also, no difference in the activity with change in alleles was observed (*P*=0.42). Data represent mean ± SEM of four independent experiments. (**B**) Relative luciferase activity of constructs containing rs72705342 with either allele ‘T’ or allele ‘C’ cloned upstream of the minimal promoter. Significant differences in reporter activities between the rs72705342 element with ‘T’ allele (203.8 ± 20.8) and ‘C’ allele (141.3 ± 14.8) or empty vector (100.0 ± 13.5) were observed with *P*-values of 0.03 and 0.001, respectively. No significant difference was observed between empty vector and construct with ‘C’ allele (*P*=0.06). Data represent mean ± SEM of four independent experiments. (**C**) Relative luciferase activity of constructs containing rs72705342 with either allele ‘C’ or allele ‘T’ upstream of *FBLN5* core promoter. Change from allele ‘C’ to ‘T’ showed a significant increase (*P*=0.02) in luciferase activity. No significant difference was observed between the empty vector and the construct with allele ‘T’ (*P*=0.15) or allele ‘C’ (*P*=0.27). Data represent mean ± SEM of three independent experiments; **P< 0.01, *P<0.05.

To evaluate the putative regulatory effect of the regions containing these SNPs, luciferase reporter assays were performed. The 29 bp regions flanking the SNPs were cloned upstream of the minimal promoter in pGL4.23 and transiently transfected into HLE B-3 cells. The cells containing constructs with either ‘G’ (*P*=0.1) or ‘A’ (*P*=0.5) allele at rs17732466 did not show any differential luciferase activity compared with empty vector. Also, no significant changes in luciferase activity were observed between the alleles (*P*=0.4) (Figure 3A). On the other hand, the alleles at rs72705342 element showed significant allele-specific changes in the luciferase activity. The presence of the protective allele ‘T’ at rs72705342 significantly increased the expression of luciferase compared with the construct with the risk allele ‘C’ (*P*=0.03) or the empty vector (*P*=0.001), the *p*-values remained significant at *P*=0.04 and *P*=0.007, respectively, after the correction for multiple pair-wise comparisons. No significant difference was observed between empty vector and construct with ‘C’ allele (Figure 3B). Further, to assess the direct effect of rs72705342 element on *FBLN5* promoter, the core promoter of *FBLN5* [24] was cloned into pGL3 basic vector and the 29 bp rs72705342 loci with either allele ‘T’ or allele ‘C’ was cloned upstream of the *FBLN5* promoter. These constructs were transiently transfected into HLE B-3 cells. The reporter activity showed that the alleles at rs72705342 showed an allele-specific effect on the *FBLN5* promoter (Figure 3C). Change in allele ‘C’ to ‘T’ at rs72705342 showed an increased luciferase activity (*P*=0.02, corrected *P*=0.03) implying an allele-specific regulatory effect of the rs72705342 element on *FBLN5* promoter.

### Risk allele ‘C’ at rs72705342 showed greater protein binding affinity compared with ‘T’ allele

**Figure 4:**
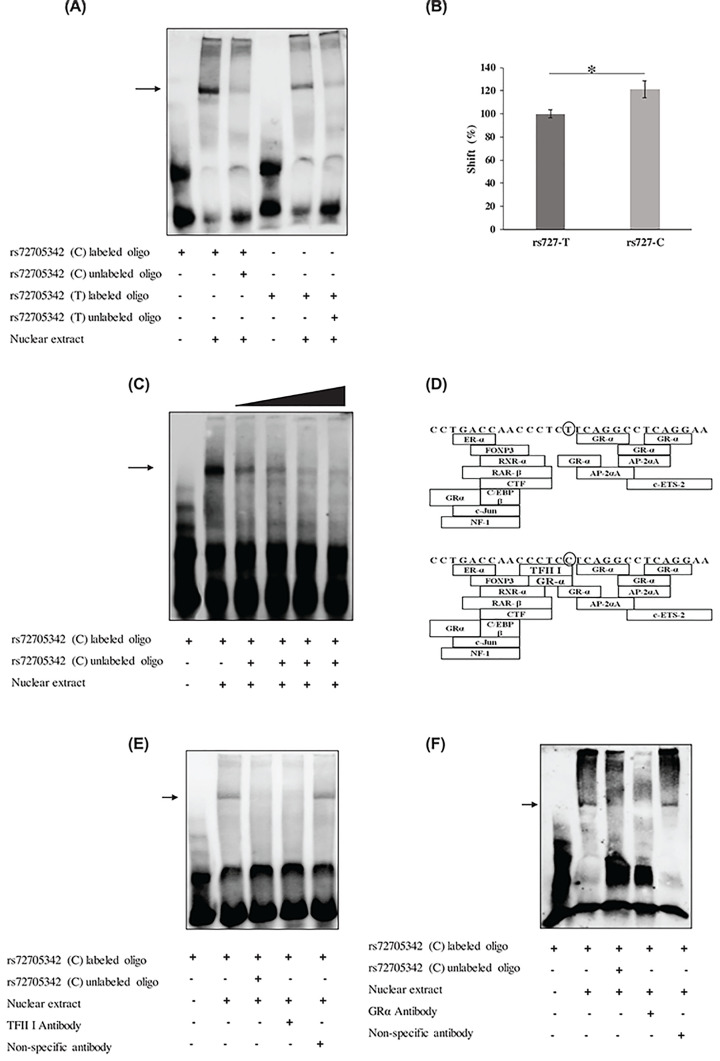
Binding of specific transcription factors to rs72705342 (**A**) EMSA using 29bp biotinylated DNA probes with rs72705342 ‘C’ or ‘T’ allele and nuclear extract from HLE B-3 cells showed specific DNA–protein complexes which vanished on competitive inhibition with respective unlabelled excess probes. (**B**) Quantitative analysis of the shifted bands relative to the unshifted bands showed allele-specific differences with significantly stronger binding of the ‘C’ allele with the nuclear protein compared with the ‘T’ allele set at 100%. Data represent mean ± SEM of four independent experiments; *P<0.05. (**C**) Competitive EMSA on rs72705342 ‘C’ robe with increasing concentrations of unlabelled excess (50-, 100-, 200- and 400-fold) showed gradual decrease in the intensity of the shift. (**D**) Allele (encircled) specific transcription factor binding at rs72705342 as predicted on PROMO software. The ‘C’ allele at rs72705342 is predicted to bind to GR-α and TFII I transcription factors, the binding of which is predicted to be lost on change in allele from ‘C’ to ‘T’. (**E**) The EMSA shows that in the presence of TFII I specific antibody, the intensity of the shift reduced which did not happen in the presence of non-specific antibody (HSF1) taken as control. (**F**) The EMSA shows that in the presence of GR-α-specific antibody, the intensity of the shift reduced which did not happen in the presence of non-specific antibody taken as control.

The electrophoretic mobility shift assays (EMSA) were performed to study specific DNA–protein interactions at rs72705342. The EMSA yielded specific shifted bands, and shifts could be competitively inhibited by excess of unlabelled oligonucleotides (Figure 4A). Quantitative analysis of the shifted bands showed greater protein binding to the sequence containing the risk allele ‘C’ compared with that containing allele ‘T’ (*P*=0.04) implying a differential transcription factor binding at rs72705342 (Figure 4B and Supplementary Figure S1). Competitive EMSA on the labelled rs72705342 ‘C’ probe with increasing concentration of unlabelled rs72705342 ‘C’ oligo (50-, 100-, 200- and 400-fold excess) showed progressive reduction of the shifted band (Figure 4C). *In silico* analysis using PROMO software predicted binding of ten transcription factors to the region flanking rs72705342 (±14 bp), i.e., glucocorticoid receptor α (GR-α), activating enhancer binding protein 2α (AP-2αA), CCAAT/enhancer-binding protein β (C/EBPβ), c-Jun, nuclear factor-1 (NF-1), estrogen receptor α (ER-α), forkhead box P3 (FOXP3), retinoid X receptor α (RXR-α), retinoic acid receptor α (RAR-α), CAAT box transcription factor (CTF) and c-ETS-2. Binding of only one transcription factor, TFII I was predicted to be affected by variation at rs72705342. Change in allele from ‘C’ to ‘T’ at rs72705342 predicted a loss of binding site for TFII I transcription factor. Also, although GR-α had binding sites in the 29 bp sequence, an additional binding site for GR-α was created in the presence of ‘C’ allele (Figure 4D). To check the binding of TFII I and GR-α to rs72705342 ‘C’, the EMSA was performed using antibodies specific to either of the transcription factors. On pre-incubating the nuclear extract with TFII I antibody, a decreased intensity of the shift was noted which did not happen in the presence of non-specific HSF1 antibody (Figure 4E). Further, as shown in Figure 4F, a reduced intensity in the shift was observed in the presence of GR-α antibody as well but not in the presence of the non-specific HSF1 antibody. However, contradictory to the *in silico* prediction, even with rs720705342 ‘T’ probe, a reduction in the intensity of shift was observed in the presence of TFII I. A decreased intensity in shift was observed with rs72705342 ‘T’ probe in the presence of GR-α antibody as well (Supplementary Figure S2A and 2B). These findings suggest that the protein–DNA complexes binding to rs72705342 element might comprise the TFII I and GR-α proteins.

## Discussion

In the present study, we investigated the association of *FBLN5* variants with PEX by Tag-SNP genotyping approach. PEX is a complex progressive multifactorial disorder of the ECM that manifests primarily in the ocular tissues. Impaired cross-linking of the ECM proteins and their subsequent aggregation is a hallmark of PEX. Association of numerous ECM proteins, such as elastin, tropoelastin, fibrillin-1, matrix metalloproteinase and their inhibitors, LOXL1 and FBLN5, with the disease pathology substantiates the debilitating effect of impaired ECM production and maintenance in development of PEX fibrils and subsequent PEX pathogenesis. FBLN5 is a matricellular scaffold protein with a crucial role to play in elastogenesis. FBLN5 interacts with integrins on the cell surface through its N-terminal domain and with LOXL1 through its C-terminal domain and brings the other ECM proteins into close proximity to facilitate elastogenesis [25]. Genetic variants in *FBLN5* and its deregulation lead to various elastinopathies such as, cutis laxa, pelvic organ prolapse (POP), Charcot-Marie-Tooth disease and AMD [14,20,21,26]. Missense substitutions leading to improper secretion of FBLN5 and reduced interaction with elastin and fibrillin-1 have been reported in recessive cutis laxa [27]. Khadzhievaa et al*.* reported association of several tag SNPs with the advanced POP [22].

We and others have reported the dysregulation of FBLN5 in PEX patients which may result in impaired elastic fiber formation, degenerative tissue alterations, and subsequent ECM protein deposition [12,28]. We also identified novel genetic association of two variants, rs7149187 in the 5’-UTR and rs929608 in the 10th intron within *FBLN5* with PEX which were, however, not found to be causal variants [12]. In the present study, we identified two intronic variants, NC_000014.9:g.91913280G>A (rs17732466:G>A) and NC_000014.9:g.91890855C>T (rs72705342:C>T) to be associated with PEXG as risk factors. However, these SNPs were not found to be associated with the early stage of PEX, PEXS. The minor alleles ‘A’ and ‘T’ at rs17732466 and rs72705342 were present in higher frequency in the controls and conferred a protective effect. The frequency of risk alleles at rs17732466 and rs72705342 was 78.0% and 82.0%, respectively, in PEXG. The 1000 Genomes Project data on Ensembl database recorded that the frequency of the risk allele ‘G’ at rs17732466 is the highest in African population (88.0%), followed by American (87.0%), East Asian (84.0%), European (75.0%), and South Asian (73.0%) populations. The frequency of the risk allele ‘C’ at rs72705342 is the highest in African population (96.0%), followed by American (88.0%), East Asian (85.0%), European (77.0%) and South Asian (74.0%) populations. We found that the frequency of risk genotype ‘CC’ at rs72705342 was the highest in PEXG (64.0%) compared with 56.0% in control and 58.0% in PEXS. Further, the haplotypes among the Tag-SNPs across the gene showed significant association with PEXG but not with PEXS. This suggests that the underlying mechanism of pathogenesis of PEXS and PEXG could be different with novel risk factors contributing to the severity of PEX in its advanced stage. Many diseases show association of genetic variants with the severe forms of the disease compared with their early stages. Variants in *CFH* and *ARMS2* were found to be more common with increasing severity of ARMD [29]. Tang et al*.* show that an intronic variant in the *PAX6* gene is associated with extreme myopia but not with mild myopia [30]. Our findings suggest that these variants in FBLN5 might contribute to development and/or progression of PEX to a severe form rather than to the onset of the disease.

As causal variants for complex disorders are also found in regions outside the coding areas of protein coding genes, the functional effect of rs17732466 and rs72705342 was assessed [31,32]. Reporter assays showed that rs72705342 has an allele-specific regulatory effect on *FBLN5* promoter. The risk allele ‘C’ at rs72705342 significantly reduced the reporter gene expression compared with allele ‘T’. This finding is supported by the eQTL data from GTeX database which shows a significant increase in FBLN5 expression in tissues from individuals with the ‘TT’ genotype compared with those with ‘CC’ genotype (Supplementary Figure S3). Further, DNA–protein binding assay showed that the sequence with ‘C’ allele has more affinity to protein binding compared with that with the ‘T’ allele implying that the allele-specific differential regulation by rs72705342 could be due to differential transcription factor binding at the alleles. This finding was supported by the *in silico* analysis which showed differential binding of two transcription factors, GR-α and TFII I with the ‘C’ allele at rs72705342. Electrophoretic mobility shift assays including the antibodies against TFII I and GR-α showed that both these proteins might bind to rs72705342 C/T. However, the extent of binding affinity of these proteins to the alleles at rs72705342 needs to be verified further through the use of CRISPR-edited cells lines followed by chromatin immunoprecipitation experiments.

This study might have a limitation in terms of the sample size included and replication of this genetic association study at a larger scale and in different cohorts is needed to gain confidence in the association of these intronic variants with PEXG and their non-association with PEXS. Also, though rs72705342 showed regulatory effect on gene expression, we observed that FBLN5 is down-regulated in the lens capsule of PEXS individuals but not PEXG. However, the association of rs72705342 with only PEXG but not PEXS suggests that this SNP might have an unknown effect on PEXG progression. It is possible that this variant could influence the expression of distal genes contributing to PEXG pathology that needs to be studied further. In conclusion, the present study elucidated genetic association of 13 tag SNPs in *FBLN5* with PEX. We identified several SNPs and haplotypes of *FBLN5* to be associated with the advanced stage of PEX, PEXG but not with the early stage of PEXS. The intronic SNP rs72705342 showed a plausible regulatory effect on *FBLN5* expression. Further, *in vivo* studies can help to understand the exact effect of these deep intronic variants and haplotypes on the progression of the disorder.

## Supplementary Material

Supplementary Figures S1-S3 and Tables S1-S3

## Data Availability

The data pertaining to this study are within the published article and its supplementary files. Any additional data will be made available by the corresponding author on reasonable request.

## Abbreviations

AMD: age-related macular degeneration
AP-2αA: activating enhancer binding protein 2α
CTF: CAAT box transcription factor
C/EBPβ: CCAAT/enhancer-binding protein β
ECM: extracellular matrix
EMSA: electrophoretic mobility shift assay
ER-α: estrogen receptor α
FBLN5: fibulin-5
FOXP3: forkhead box P3
GRα: glucocorticoid receptor α
HLE B-3: Human lens epithelial B3 cell
HWE: Hardy–Weinberg equilibrium
IOP: intraocular pressure
LC: lens capsule
LD: linkage disequilibrium
LOXL1: lysyl oxidase-like 1
MAF: minor allele frequency
NF-1: nuclear factor 1
PEX: pseudoexfoliation
PEXG: pseudoexfoliation glaucoma
PEXS: pseudoexfoliation syndrome
POP: pelvic organ prolapse
RAR-α: retinoic acid receptor α
ROS: reactive oxygen species
RXR-α: retinoid X receptor α
SNP: single-nucleotide polymorphism

## References

[1] Plateroti P., Plateroti A.M., Abdolrahimzadeh S. et al. (2015) Pseudoexfoliation syndrome and pseudoexfoliation glaucoma: a review of the literature with updates on surgical management. J. Ophthalmol. 2015, 1–9 10.1155/2015/370371 26605078 PMC4641922

[2] Gottanka J., Flügel-Koch C., Martus P. et al. (1997) Correlation of pseudoexfoliative material and optic nerve damage in pseudoexfoliation syndrome. Invest. Ophthalmol. Vis. Sci. 38, 2435–2446 9375560

[3] Ritch R. (1994) Exfoliation syndrome and occludable angles. Transactions of the American Ophthalmological Society, pp. 845–944, American Ophthalmological Society PMC1298528 7886885

[4] Hohberger B., Schlötzer-Schrehard U., Mardin C. et al. (2021) Inhibitory and agonistic autoantibodies directed against the β2-adrenergic receptor in pseudoexfoliation syndrome and glaucoma. Front. Neurosci. 15, 1–11 10.3389/fnins.2021.676579 PMC8377674 34421514

[5] Krishnadas R., Nirmalan P.K., Ramakrishnan R. et al. (2003) Pseudoexfoliation in a rural population of southern India: The Aravind Comprehensive Eye Survey. Am. J. Ophthalmol. 135, 830–837 10.1016/S0002-9394(02)02271-7 12788123

[6] Arvind H., Raju P., Paul P.G. et al. (2003) Pseudoexfoliation in south India. Br. J. Ophthalmol. 87, 1321–1323 10.1136/bjo.87.11.1321 14609823 PMC1771878

[7] Olawoye O.O., Pasquale L.R. and Ritch R. (2014) Exfoliation syndrome in sub-Saharan Africa. Int. Ophthalmol. 34, 1165–1173 10.1007/s10792-014-9953-5 24844849

[8] Rao R.Q., Arain T.M. and Ahad M.A. (2006) The prevalence of pseudoexfoliation syndrome in Pakistan. Hospital based study. BMC Ophthalmol. 6, 1–5 10.1186/1471-2415-6-27 16792800 PMC1513600

[9] Zenkel M. and Schlötzer-Schrehardt U. (2014) The composition of exfoliation material and the cells involved in its production. J. Glaucoma 23, S12–S14 10.1097/IJG.0000000000000123 25275897

[10] Padhy B., Nanda G.G., Chowdhury M. et al. (2014) Role of an extracellular chaperone, clusterin in the pathogenesis of pseudoexfoliation syndrome and pseudoexfoliation glaucoma. Exp. Eye Res. 127, 69–76 10.1016/j.exer.2014.07.005 25057782

[11] Challa P. and Johnson W.M. (2018) Composition of exfoliation material. J. Glaucoma 27, S29–S31 10.1097/IJG.0000000000000917 29965899

[12] Padhy B., Kapuganti R.S., Hayat B. et al. (2019) De novo variants in an extracellular matrix protein coding gene, fibulin-5 (FBLN5) are associated with pseudoexfoliation. Eur. J. Hum. Genet. 27, 1858–1866 10.1038/s41431-019-0482-6 31358954 PMC6871134

[13] Yanagisawa H., Schluterman M.K. and Brekken R.A. (2009) Fibulin-5, an integrin-binding matricellular protein: its function in development and disease. J. Cell Commun. Signal 3, 337–347 10.1007/s12079-009-0065-3 19798595 PMC2778585

[14] Jung H.J., Jeon M.J., Yim G.W. et al. (2009) Changes in expression of fibulin-5 and lysyl oxidase-like 1 associated with pelvic organ prolapse. Eur. J. Obstetrics Gynecol. Reproduct. Biol. 145, 117–122 10.1016/j.ejogrb.2009.03.026 19450918

[15] Wirostko B.M., Curtin K., Ritch R. et al. (2016) Risk for exfoliation syndrome in women with pelvic organ prolapse: a UTAH project on exfoliation syndrome (UPEXS) study. JAMA Ophthalmol. 134, 1255–1262 10.1001/jamaophthalmol.2016.3411 27632406

[16] Schumacher S., Schlötzer-Schrehardt U., Martus P. et al. (2001) Pseudoexfoliation syndrome and aneurysms of the abdominal aorta. Lancet 357, 359–360 10.1016/S0140-6736(00)03645-X 11211000

[17] Lotery A.J., Baas D., Ridley C. et al. (2006) Reduced secretion of fibulin 5 in age-related macular degeneration and cutis laxa. Hum. Mutat. 27, 568–574 10.1002/humu.20344 16652333 PMC1828612

[18] Orriols M., Varona S., Martí-Pàmies I. et al. (2016) Down-regulation of Fibulin-5 is associated with aortic dilation: role of inflammation and epigenetics. Cardiovasc. Res. 110, 431–442 10.1093/cvr/cvw082 27089918

[19] Kozobolis V.P., Detorakis E.T., Tsilimbaris M.K. et al. (1999) Correlation between age-related macular degeneration and pseudoexfoliation syndrome in the population of Crete (Greece). Arch. Ophthalmol. 117, 664–669 10.1001/archopht.117.5.664 10326966

[20] Stone E.M., Braun T.A., Russell S.R. et al. (2004) Missense variations in the fibulin 5 gene and age-related macular degeneration. N. Engl. J. Med. 351, 346–353 10.1056/NEJMoa040833 15269314

[21] Claus S., Fischer J., Mégarbané H. et al. (2008) A p.C217R mutation in fibulin-5 from cutis laxa patients is associated with incomplete extracellular matrix formation in a skin equivalent model. J. Invest. Dermatol. 128, 1442–1450 10.1038/sj.jid.5701211 18185537

[22] Khadzhieva M.B., Kamoeva S.V., Chumachenko A.G. et al. (2014) Fibulin-5 (FBLN5) gene polymorphism is associated with pelvic organ prolapse. Maturitas 78, 287–292 10.1016/j.maturitas.2014.05.003 24917111

[23] Andley U.P., Rhim J.S., Chylack L.T. et al. (1994) Propagation and immortalization of human lens epithelial cells in culture. Invest. Ophthalmol. Vis. Sci. 35, 3094–3102 8206728

[24] Kuang P.P., Joyce-Brady M., Zhang X.H. et al. (2006) Fibulin-5 gene expression in human lung fibroblasts is regulated by TGF-β and phosphatidylinositol 3-kinase activity. Am. J. Physiol. Cell Physiol. 291, C1412–C1421 10.1152/ajpcell.00087.2006 16837650

[25] Kobayashi N., Kostka G., Garbe J.H.O. et al. (2007) A comparative analysis of the fibulin protein family: biochemical characterization, binding interactions, and tissue localization. J. Biol. Chem. 282, 11805–11816 10.1074/jbc.M611029200 17324935

[26] Auer-grumbach M., Weger M., Fink-puches R. et al. (2012) Europe PMC Funders Group fibulin -5 mutations link inherited neuropathies, age-related macular degeneration and hyperelastic skin. 134, 1839–1852 21576112 10.1093/brain/awr076 PMC3272386

[27] Hu Q., Reymond J.L., Pinel N. et al. (2006) Inflammatory destruction of elastic fibers in acquired cutis laxa is associated with missense alleles in the elastin and fibulin-5 genes. J. Invest. Dermatol. 126, 283–290 10.1038/sj.jid.5700047 16374472

[28] Rebecca M., Sripriya K., Bharathselve M. et al. (2022) Increased desmosine in the lens capsules is associated with augmented elastin turnover in pseudoexfoliation syndrome. Exp. Eye Res. 215, 108898 10.1016/j.exer.2021.108898 34929161

[29] Farwick A., Dasch B., Weber B.H.F. et al. (2009) Variations in five genes and the severity of age-related macular degeneration: Results from the Muenster aging and retina study. Eye 23, 2238–2244 10.1038/eye.2008.426 19169232

[30] Tang S.M., Ma L., Lu S.Y. et al. (2018) Association of the PAX6 gene with extreme myopia rather than lower grade myopias. Br. J. Ophthalmol. 102, 570–574 10.1136/bjophthalmol-2017-311327 29436400

[31] Padhy B., Hayat B., Nanda G.G. et al. (2017) Pseudoexfoliation and Alzheimer's associated CLU risk variant, rs2279590, lies within an enhancer element and regulates CLU, EPHX2 and PTK2B gene expression. Hum. Mol. Genet. 26, 4519–4529 10.1093/hmg/ddx329 28973302

[32] Berner D., Hoja U., Zenkel M. et al. (2019) The protective variant rs7173049 at LOXL1 locus impacts on retinoic acid signaling pathway in pseudoexfoliation syndrome. Hum. Mol. Genet. 28, 2531–2548 10.1093/hmg/ddz075 30986821 PMC6644155

