# Supplementary material for: Genetic variants and haplotypes in fibulin-5 (FBLN5) are associated with pseudoexfoliation glaucoma but not with pseudoexfoliation syndrome
Source: Biosci Rep. 2023 Mar 2;43(3):BSR20221622. doi: 10.1042/BSR20221622 (PMC9995586; doi:10.1042/BSR20221622)
Supplement: Supplementary Figures S1-S3 and Tables S1-S3 [file BSR-2022-1622_supp.pdf]

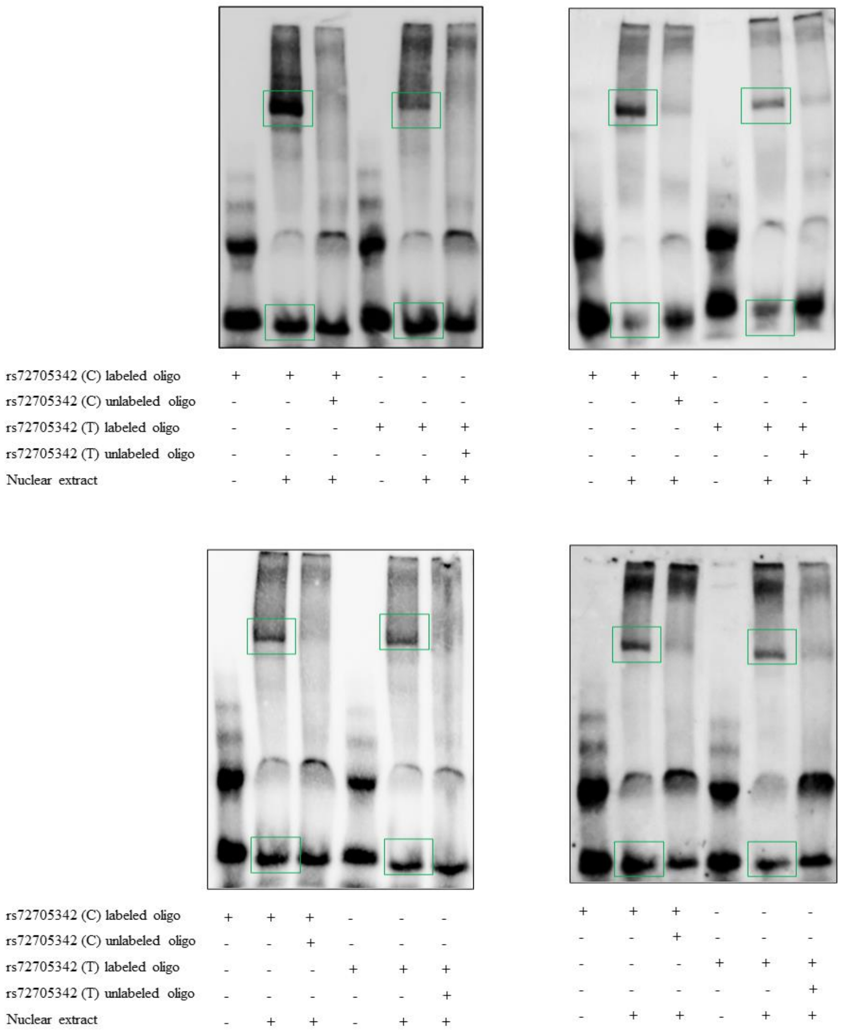

Supplementary Figure 1. EMSA blots showing binding of rs72705342 element with nuclear proteins of HLE B-3 cells. The boxes indicate the bands whose densitometric values were used to calculate the % shift.

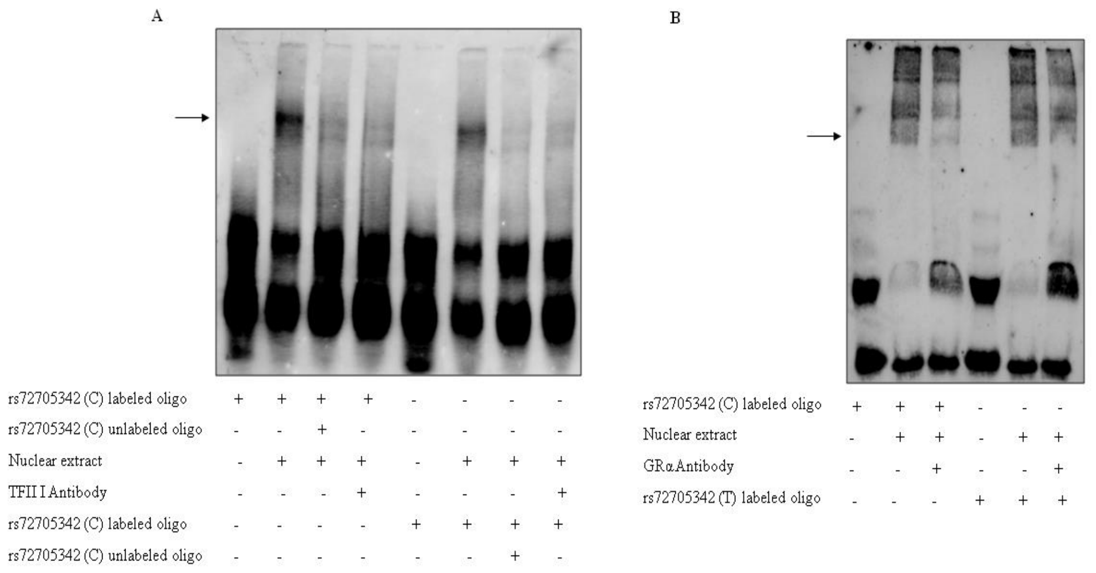

Supplementary Figure 2. A. EMSA blots showing reduction in the intensity of the shift from interaction of rs72705342 'C' and 'T' probes with nuclear extract in the presence of TFII I antibody. **B.** EMSA blots showing reduction in the intensity of the shift from interaction of rs72705342 'C' and 'T' probes with nuclear extract in the presence of GR-α antibody.

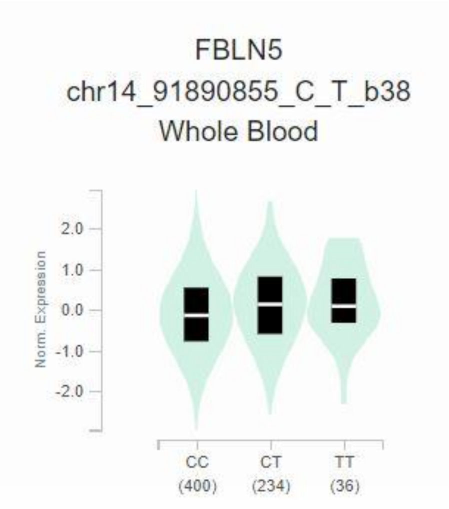

Supplementary Figure 3. rs72705342 is an eQTL for *FBLN*5 expression The eQTL violin plot obtained from GTex portal shows that tissue with homozygous alternate genotype ‘TT’ shows increased *FBLN5* expression (p<0.001) compared to the homozygous reference genotype ‘CC’.

Supplementary Table 1. Tag SNPs in *FBLN5* (Chr 14, hg38 build) chosen for this study

Variant	dbSNP ID	Location on gene
NC_000014.9:g.91943755T>C	rs12432450:T>C	Intron 1
NC_000014.9:g.91926682C>T	rs8012648:C>T	Intron 4
NC_000014.9:g.91913280G>A	rs17732466:G>A	Intron 4
NC_000014.9:g.91911458G>A	rs12589592:G>A	Intron 4
NC_000014.9:g.91904479G>T	rs2498835:G>T	Intron 4
NC_000014.9:g.91902891C>G	rs2267997:C>G	Intron 4
NC_000014.9:g.91897442T>C	rs917908:T>C	Intron 4
NC_000014.9:g.91896083T>C	rs2244158:T>C	Intron 4
NC_000014.9:g.91892576C>T	rs2243400:C>T	Intron 5
NC_000014.9:g.91891905G>C	rs2267995:G>C	Intron 5
NC_000014.9:g.91890855C>T	rs72705342:C>T	Intron 6
NC_000014.9:g.91885243A>C	rs2498841:A>C	Intron 7

Supplementary Table 2. List of oligos used in the study

S. No.	ID	Purpose	Sequence (5'→3')
1	rs72705342 'C'	Luciferase assay	Top: CTTCCTGAGGCCTGAGGAGGGTTGGTCAGGC<br>Bot: TCGAGCCTGACCAACCCTCCTCAGGCCTCAGGAAGGTAC
2	rs72705342 'T'	Luciferase assay	Top: CTTCCTGAGGCCTGAAGAGGGTTGGTCAGGC<br>Bot: TCGAGCCTGACCAACCCTCTTCAGGCCTCAGGAAGGTAC
3	rs17732466 'G'	Luciferase assay	Top: CATCCTCCAAAATATTCAGGCATGATATTCC<br>Bot: TCGAGGAATATCATGCCTGAATATTTTGGAGGATGGTAC
4	rs17732466 'A'	Luciferase assay	Top: CATCCTCCAAAATATTTAGGCATGATATTCC<br>Bot: TCGAGGAATATCATGCCTAAATATTTTGGAGGATGGTAC
5	*FBLN5* promoter	Luciferase assay	FP: CACGAAGCTTTTTCTAGTCCCTGGAGCTGCG<br>RP: CACTCCATGGGTCCAAGACGCGCGAGGA
7	rs72705342 'C' labelled	EMSA	S: TTCCTGAGGCCTGAGGAGGGTTGGTCAGG<br>A: CCTGACCAACCCTCCTCAGGCCTCAGGAA
8	rs72705342 'T' labelled	EMSA	S: TTCCTGAGGCCTGAAGAGGGTTGGTCAGG<br>A: CCTGACCAACCCTCTTCAGGCCTCAGGAA

Supplementary Table 3. Haplotype distribution of *FBLN5* variants in block 2 of controls, PEXS and PEXG

Haplotype (rs917908-rs2267997-rs2498835)	Freq. in control	Freq. in PEXS	Freq. in PEXG	Control vs. PEXS		Control vs. PEXG	
				OR (95% CI)	p-value	OR (95% CI)	p-value
T-G-G	0.32	0.30	0.36	1.11 (0.81-1.53)	0.50	0.83 (0.58-1.19)	0.32
T-C-G	0.31	0.32	0.33	0.97 (0.71-1.33)	0.87	0.93 (0.64-1.34)	0.69
T-G-T	0.24	0.24	0.18	1.02 (0.72-1.44)	0.89	1.40 (0.91-2.14)	0.11
C-G-T	0.11	0.12	0.10	0.90 (0.56-1.43)	0.65	1.06 (0.61-1.85)	0.80

CI: confidence interval, OR: odds ratio, PEXS: pseudoexfoliation syndrome, PEXG: pseudoexfoliation glaucoma
